# What is CRISPR/Cas9?

**DOI:** 10.1136/archdischild-2016-310459

**Published:** 2016-04-08

**Authors:** Melody Redman, Andrew King, Caroline Watson, David King

**Affiliations:** 1 HYMS Centre for Education Development (CED), Hull York Medical School, University of York, York, UK; 2 Molecular Haematology Unit, MRC Weatherall Institute of Molecular Medicine, University of Oxford, John Radcliffe Hospital, Oxford, UK; 3 Department of Haematology, Oxford University NHS Foundation Trust, Churchill Hospital, Oxford, UK; 4 Academic Unit of Child Health, Sheffield Children's Hospital, Sheffield, UK

**Keywords:** CRISPR/cas9, gene editing, children, genome engineering

## Introduction

Clustered regularly interspaced palindromic repeats (CRISPR)/Cas9 is a gene-editing technology causing a major upheaval in biomedical research. It makes it possible to correct errors in the genome and turn on or off genes in cells and organisms quickly, cheaply and with relative ease. It has a number of laboratory applications including rapid generation of cellular and animal models, functional genomic screens and live imaging of the cellular genome.^1^ It has already been demonstrated that it can be used to repair defective DNA in mice curing them of genetic disorders,^2^ and it has been reported that human embryos can be similarly modified.^3^ Other potential clinical applications include gene therapy, treating infectious diseases such as HIV and engineering autologous patient material to treat cancer and other diseases. In this review we will give an overview of CRISPR/Cas9 with an emphasis on how it may impact on the specialty of paediatrics. Although it is likely to have a significant effect on paediatrics through its impact in the laboratory, here we will concentrate on its potential clinical applications. We will also describe some of the difficulties and ethical controversies associated with this novel technology.

## Overview of CRISPR/Cas9

**Figure 1:**
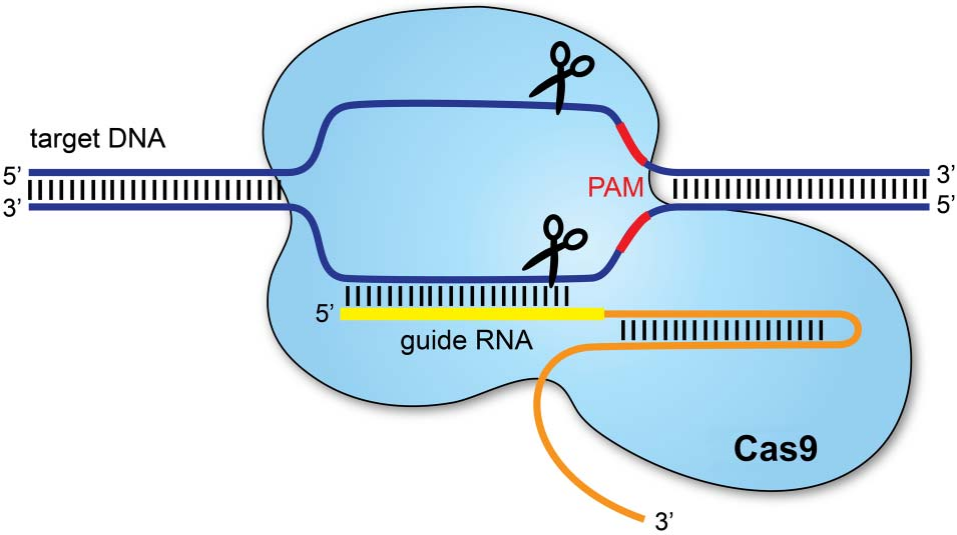
The CRISPR/Cas9 system.^1^ Clustered regularly interspaced palindromic repeats (CRISPR) refers to sequences in the bacterial genome. They afford protection against invading viruses, when combined with a series of CRISPR-associated (Cas) proteins. Cas9, one of the associated proteins, is an endonuclease that cuts both strands of DNA. Cas9 is directed to its target by a section of RNA. This can be synthesised as a single strand called a synthetic single guide RNA (sgRNA); the section of RNA which binds to the genomic DNA is 18–20 nucleotides. In order to cut, a specific sequence of DNA of between 2 and 5 nucleotides (the exact sequence depends upon the bacteria which produces the Cas9) must lie at the 3’ end of the guide RNA: this is called the protospacer adjacent motif (PAM). Repair after the DNA cut may occur via two pathways: non-homologous end joining, typically leading to a random insertion/deletion of DNA, or homology directed repair where a homologous piece of DNA is used as a repair template. It is the latter which allows precise genome editing: the homologous section of DNA with the required sequence change may be delivered with the Cas9 nuclease and sgRNA, theoretically allowing changes as precise as a single base-pair.

CRISPR/Cas9 is a gene-editing technology which involves two essential components: a guide RNA to match a desired target gene, and Cas9 (CRISPR-associated protein 9)—an endonuclease which causes a double-stranded DNA break, allowing modifications to the genome (see figure 1).

## Potential clinical uses

### Correction of genetic disorders

One of the most exciting applications of CRISPR/Cas9 is its potential use to treat genetic disorders caused by single gene mutations. Examples of such diseases include cystic fibrosis (CF), Duchenne's muscular dystrophy (DMD) and haemoglobinopathies. The approach so far has currently only been validated in preclinical models, but there is hope it can soon be translated to clinical practice.

Schwank *et al* used CRISPR/Cas9 to investigate the treatment of CF. Using adult intestinal stem cells obtained from two patients with CF, they successfully corrected the most common mutation causing CF in intestinal organoids. They demonstrated that once the mutation had been corrected, the function of the CF transmembrane conductor receptor (CFTR) was restored.^4^

Another disease in which CRISPR/Cas9 has been investigated is DMD. Tabebordbar *et al* recently used adeno-associated virus (AAV) delivery of CRISPR/Cas9 endonucleases to recover dystrophin expression in a mouse model of DMD, by deletion of the exon containing the original mutation. This produces a truncated, but still functional protein. Treated mice were shown to partially recover muscle functional deficiencies.^5^ Significantly, it was demonstrated that the dystrophin gene was edited in muscle stem cells which replenish mature muscle tissue. This is important to ensure any therapeutic effects of CRISPR/Cas9 do not fade over time. Two similar studies have described using the CRISPR/Cas9 system in vivo to increase expression of the dystrophin gene and improve muscle function in mouse models of DMD.^6^ ^7^ Other studies have used CRISPR/Cas9 to target duplication of exons in the human dystrophin gene in vitro and have shown that this approach can lead to production of full-length dystrophin in the myotubules of an individual with DMD.^8^

CRISPR/Cas9 could also be used to treat haemoglobinopathies. Canver *et al*^9^ recently showed *BCL11A* enhancer disruption by CRISPR/Cas9 could induce fetal haemoglobin in both mice and primary human erythroblast cells. In the future such an approach could allow fetal haemoglobin to be expressed in patients with abnormal adult haemoglobin. This would represent a novel therapeutic strategy in patients with diseases such as sickle cell disease or thalassaemias. Knock-in of a fully functional β-globin gene is much more challenging, which is the reason for this somewhat unusual approach.

### Treatment of HIV

Another potential clinical application of CRISPR/Cas9 is to treat infectious diseases, such as HIV. Although antiretroviral therapy provides an effective treatment for HIV, no cure currently exists due to permanent integration of the virus into the host genome. Hu *et al* showed the CRISPR/Cas9 system could be used to target HIV-1 genome activity. This inactivated HIV gene expression and replication in a variety of cells which can be latently infected with HIV, without any toxic effects. Furthermore, cells could also be immunised against HIV-1 infection. This is a potential therapeutic advance in overcoming the current problem of how to eliminate HIV from infected individuals. After further refinement, the authors suggest their findings may enable gene therapies or transplantation of genetically altered bone marrow stem cells or inducible pluripotent stem cells to eradicate HIV infection.^10^

### Engineering somatic cells ex vivo to treat malignancy or other diseases

There has been increasing interest in the possibility of using CRISPR/Cas9 to modify patient-derived T-cells and stem/progenitor cells which can then be reintroduced into patients to treat disease. This approach may overcome some of the issues associated with how to efficiently deliver gene editing to the right cells.

T-cell genome engineering has already shown success in treating haematological malignancies and has the potential to treat solid cancers, primary immune deficiencies and autoimmune diseases. Genetic manipulation of T-cells has previously been inefficient. However, Schumann *et al* recently reported a more effective approach in human CD4^+^ T-cells based on the CRISPR/Cas9 system. Their technique allowed experimental and therapeutic knock-out and knock-in editing of the genome in primary human T-cells. They demonstrated T-cells could be manipulated to prevent expression of the protein PD-1, which other work has shown may allow T-cells to target solid cancers.^11^

There is also interest in using CRISPR/Cas9-mediated genome editing in pluripotent stem cells or primary somatic stem cells to treat disease. For example Xie *et al*^12^ showed the mutation causing β-thalassaemia could be corrected in human induced pluripotent stem cells ex vivo. They suggest that in the future such an approach could provide a source of cells for bone marrow transplantation to treat β-thalassaemia and other similar monogenic diseases.

## Limitations

A number of challenges remain before the potential of CRISPR/Cas9 can be translated to effective treatments at the bedside. A particular issue is how to deliver gene editing to the right cells, especially if the treatment is to be delivered in vivo. To safely deliver Cas9-nuclease encoding genes and guide RNAs in vivo without any associated toxicity, a suitable vector is needed. AAV has previously been a favoured option for gene delivery.^1^ However, this delivery system may be too small to allow efficient transduction of the Cas9 gene.^1^ A smaller Cas9 gene could be used, but this has additional implications on efficacy.^1^ A number of other non-viral delivery systems are under investigation and this process requires further optimisation.

Another significant concern is the possibility of off-target effects on parts of the genome separate from the region being targeted. Unintentional edits of the genome could have profound long-term complications for patients, including malignancy. The concentration of the Cas9 nuclease enzyme and the length of time Cas9 is expressed are both important when limiting off-target activity.^1^ Although recent modifications in the nuclease have increased specificity, further work is required to minimise off-target effects and to establish the long-term safety of any treatment.

The therapeutic applications of CRISPR/Cas9 considered in this article have predominantly been directed at somatic cells. A particularly controversial issue surrounding CRISPR/Cas9 is that of gene editing in embryos. It has already been shown that CRISPR/Cas9 technology can alter the genome of human embryos^3^ which theoretically could prove useful in the preimplantation treatment of genetic diseases. However, any genetic modification of the germline would be permanent and the long-term consequences are unclear. Many oppose germline modification under any circumstances, reasoning that an eventual consequence could be non-therapeutic genetic enhancement.^13^ It is clear that the ethical boundaries, within which CRISPR/Cas9 can be used, remain to be fully determined.

**Clinical bottom line**

- CRISPR/Cas9 technology has the potential to revolutionise the treatment of many paediatric conditions.
- A number of practical and ethical challenges must be overcome before this potential can be realised at the bedside.
